# Intervention against hypertension in the next generation programmed by developmental hypoxia

**DOI:** 10.1371/journal.pbio.2006552

**Published:** 2019-01-22

**Authors:** Kirsty L. Brain, Beth J. Allison, Youguo Niu, Christine M. Cross, Nozomi Itani, Andrew D. Kane, Emilio A. Herrera, Katie L. Skeffington, Kimberley J. Botting, Dino A. Giussani

**Affiliations:** 1 Department of Physiology, Development, and Neuroscience, University of Cambridge, Cambridge, United Kingdom; 2 Cambridge Cardiovascular Strategic Research Initiative, Cambridge, United Kingdom; University of Pittsburgh, United States of America

## Abstract

Evidence derived from human clinical studies and experimental animal models shows a causal relationship between adverse pregnancy and increased cardiovascular disease in the adult offspring. However, translational studies isolating mechanisms to design intervention are lacking. Sheep and humans share similar precocial developmental milestones in cardiovascular anatomy and physiology. We tested the hypothesis in sheep that maternal treatment with antioxidants protects against fetal growth restriction and programmed hypertension in adulthood in gestation complicated by chronic fetal hypoxia, the most common adverse consequence in human pregnancy. Using bespoke isobaric chambers, chronically catheterized sheep carrying singletons underwent normoxia or hypoxia (10% oxygen [O_2_]) ± vitamin C treatment (maternal 200 mg.kg^−1^ IV daily) for the last third of gestation. In one cohort, the maternal arterial blood gas status, the value at which 50% of the maternal hemoglobin is saturated with oxygen (P_50_), nitric oxide (NO) bioavailability, oxidative stress, and antioxidant capacity were determined. In another, naturally delivered offspring were raised under normoxia until early adulthood (9 months). Lambs were chronically instrumented and cardiovascular function tested in vivo. Following euthanasia, femoral arterial segments were isolated and endothelial function determined by wire myography. Hypoxic pregnancy induced fetal growth restriction and fetal oxidative stress. At adulthood, it programmed hypertension by enhancing vasoconstrictor reactivity and impairing NO-independent endothelial function. Maternal vitamin C in hypoxic pregnancy improved transplacental oxygenation and enhanced fetal antioxidant capacity while increasing NO bioavailability, offsetting constrictor hyper-reactivity and replenishing endothelial function in the adult offspring. These discoveries provide novel insight into mechanisms and interventions against fetal growth restriction and adult-onset programmed hypertension in an animal model of complicated pregnancy in a species of similar temporal developmental milestones to humans.

## Author summary

Adverse conditions during pregnancy can increase the cardiovascular risk of the adult offspring. However, the mechanisms underlying these effects remain unclear, precluding the identification of candidate therapy. In this interventional study in sheep, a species of similar temporal developmental milestones to humans, we adopt an integrative approach, combining studies in vivo with those at the isolated organ, cellular, and molecular levels to investigate consequences of suboptimal pregnancy on the offspring at two stages of life: in the near-term fetus and in the adult. We show that developmental hypoxia, the most common outcome in human suboptimal pregnancy, slows fetal growth and programs high blood pressure in the sheep adult offspring. Maternal treatment with vitamin C in hypoxic pregnancy restored fetal growth and protected against adult-onset hypertension by improving transplacental oxygen delivery, enhancing fetal antioxidant capacity, and increasing the bioavailability of nitric oxide (NO) in the sheep adult offspring. Our discoveries highlight that when considering strategies to reduce the overall burden of heart disease, a much greater attention to prevention rather than treatment is required. Treatment should start as early as possible during the developmental trajectory, rather than waiting until adulthood when the disease process has become irreversible.

## Introduction

Cardiovascular disease kills 1 in 3 people [1]. The annual costs for patient care and lost workforce due to heart disease are over US$130 billion in the United States and Canada [2] and over £30 billion in the United Kingdom [3]. Therefore, cardiovascular disease is a significant problem imposing a substantial burden on every nation’s health and wealth [4]. It is widely accepted that our genes interact with traditional lifestyle factors, such as smoking, obesity, and/or a sedentary lifestyle, to promote an increased risk of cardiovascular disease [5]. It is also established that the gene–environment interaction early in life may be just as, if not more, important in “programming” heart health and heart disease [6–8]. Evidence from human sibling-pair studies suggests that these relationships are causal, that they occur independently of genotype, and that they are significantly influenced by the quality of the intrauterine environment during pregnancy [9–12]. For instance, studies in Pima Indians showed a greater prevalence of type 2 diabetes in siblings born from pregnancies during which the mother had gestational diabetes compared to those whose mother did not [9]. Bariatric surgery to decrease the weight of obese women reduced the risk of obesity, insulin resistance, and raised blood pressure in children born after surgery compared to those born before surgery [10–12]. Therefore, these studies highlight a disproportionate risk of disease in offspring born from the same mother but under different in utero conditions, providing strong evidence in humans that the environment experienced during this critical period of development directly influences long-term cardiovascular health.

One of the most common outcomes of complicated pregnancy in humans is chronic fetal hypoxia leading to reduced fetal growth, as can occur during placental insufficiency, preeclampsia, or inflammatory conditions during pregnancy, such as in chorioamnionitis, gestational diabetes, or maternal obesity [13,14]. In humans, low birth weight is related to poor neonatal outcome [15] and endothelial dysfunction [16] and high blood pressure at adulthood [17]. In turn, increased blood pressure is associated with an increased risk of cardiovascular disease [18], with this risk being greatest in those who were smallest at birth but with the most accelerated weight gain in childhood [19]. To date, there is no cure for pregnancy complicated by chronic fetal hypoxia to protect against fetal growth restriction or programmed cardiovascular dysfunction in the offspring. Treatment options are restricted to monitoring surrogate measures of fetal hypoxia and fetal growth, ultimately ending up in elective delivery of the offspring [20]. This highlights the need for experimental studies addressing underlying mechanisms to identify plausible intervention.

Studies in animal models of adverse pregnancy have confirmed causality, reporting that oxidative stress during complicated pregnancy, including one involving chronic fetal hypoxia, may be a potential underlying mechanism [8,21–31]. Chronic fetal hypoxia is a powerful stimulus for reactive oxygen species (ROS) generation [8]. Under physiologic conditions, ROS are important mediators of a wide variety of cell functions, for instance, via signaling or by interacting with nitric oxide (NO) to provide a vascular oxidant tone [32–34]. However, excessive ROS and/or a fall in antioxidant defenses can lead to cellular oxidative stress and a fall in the bioavailability of NO, predisposing to cardiovascular dysfunction [8,31]. A small cluster of investigations including our own, mostly through studies of isolated hearts and vessels or echocardiography in rodents, has provided evidence for possible intervention with maternal treatment with antioxidants to protect against the ill effects of hypoxic pregnancy on the offspring [28–31,35,36]. However, when working with animal models of cardiovascular dysfunction before birth, the temporal profile of cardiovascular development between species is a highly important consideration for successful interventional translation to the human clinical situation. Rodents are altricial species, in which cardiovascular maturation continues past birth, becoming completed by the second week of postnatal life [28]. In contrast, sheep and humans share similar prenatal tempos of cardiovascular development [28] and some breeds of sheep, like Welsh Mountain, give birth primarily to singleton lambs of similar weight to term human babies. To date, no study has addressed maternal antioxidant intervention to protect against systemic cardiovascular dysfunction in the adult offspring in a human translational model of hypoxic pregnancy, such as in sheep, which additionally permits detailed cardiovascular analysis of in vivo mechanisms of action.

**Fig 1:**
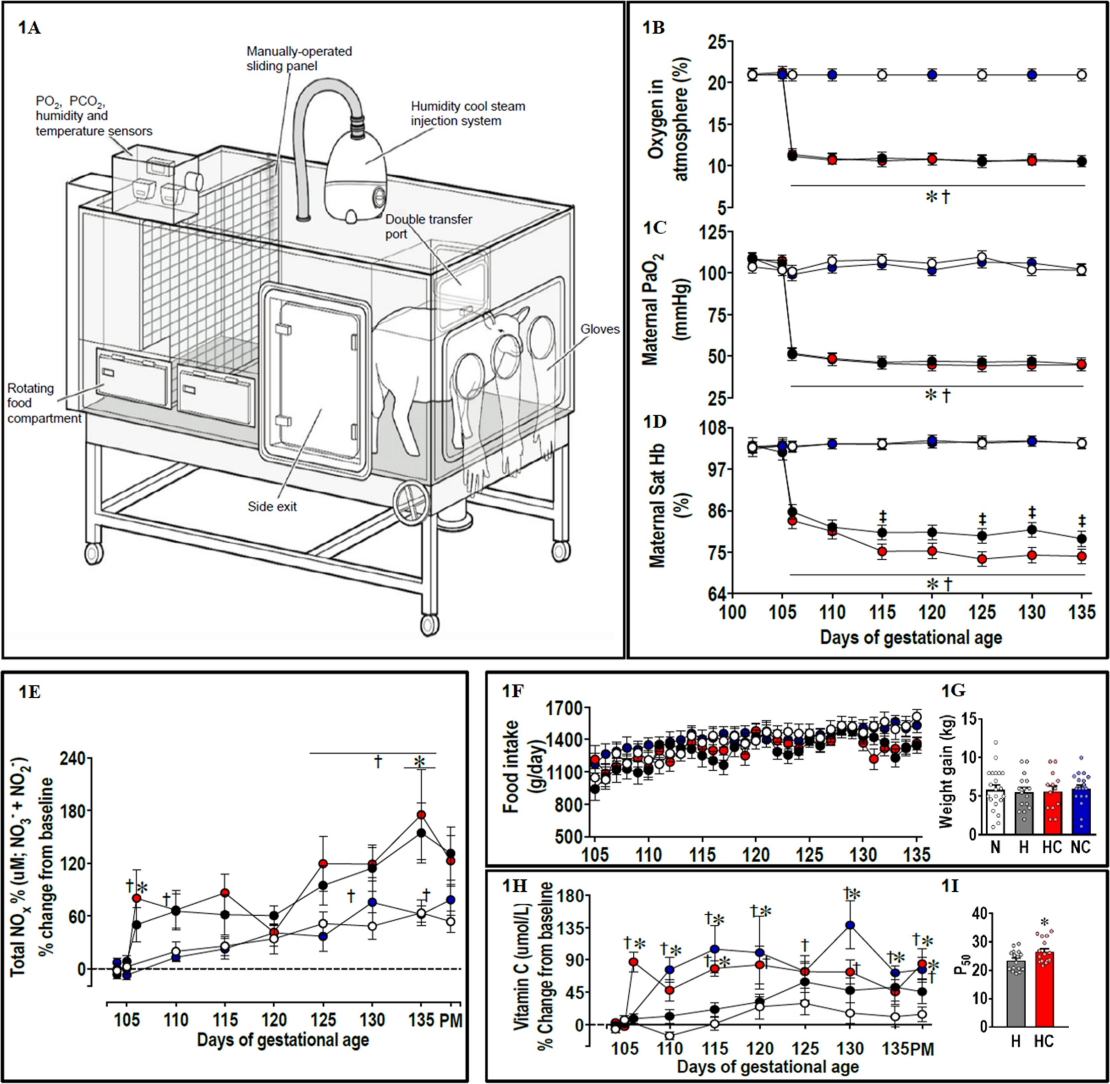
Maternal data. 1A. Isobaric hypoxic chamber; maternal variables measured during the experimental period are the following: 1B, maternal atmospheric oxygen exposure; 1C, arterial blood partial pressure of oxygen (PaO_2_); 1D, Sat Hb; 1E, plasma NOx during the experimental period and at PM; 1F, maternal daily food consumption; 1G, maternal weight gain during the experimental period; 1H, plasma vitamin C during the experimental period and at PM, and 1I, the P_50_ value of the relationship between the maternal PaO_2_ plotted against the Sat Hb. Values are mean ± SEM for all data. Groups are N (open symbols, *n* = 7–21), H (black/grey symbols, *n* = 7–22), HC (red symbols, *n* = 6–19), and NC (blue symbols, *n* = 9–18). Significant (*P* < 0.05) differences are the following: *versus normoxia, † versus baseline, ‡H versus HC, and two-way repeated-measures ANOVA with posthoc Tukey test (1B–1F and 1H) or Student *t* test for unpaired data (1I). H, hypoxia; HC, hypoxia with vitamin C; N, normoxia; NC, normoxia with vitamin C; NOx, plasma concentrations of total NO_3_^−^ + NO_2_^−^; P_50,_ PaO_2_ value at which 50% of hemoglobin is saturated with oxygen; PaO_2_, arterial blood partial pressure of oxygen; PM, post mortem; Sat Hb, arterial blood percentage saturation of hemoglobin with oxygen.

**Fig 2:**
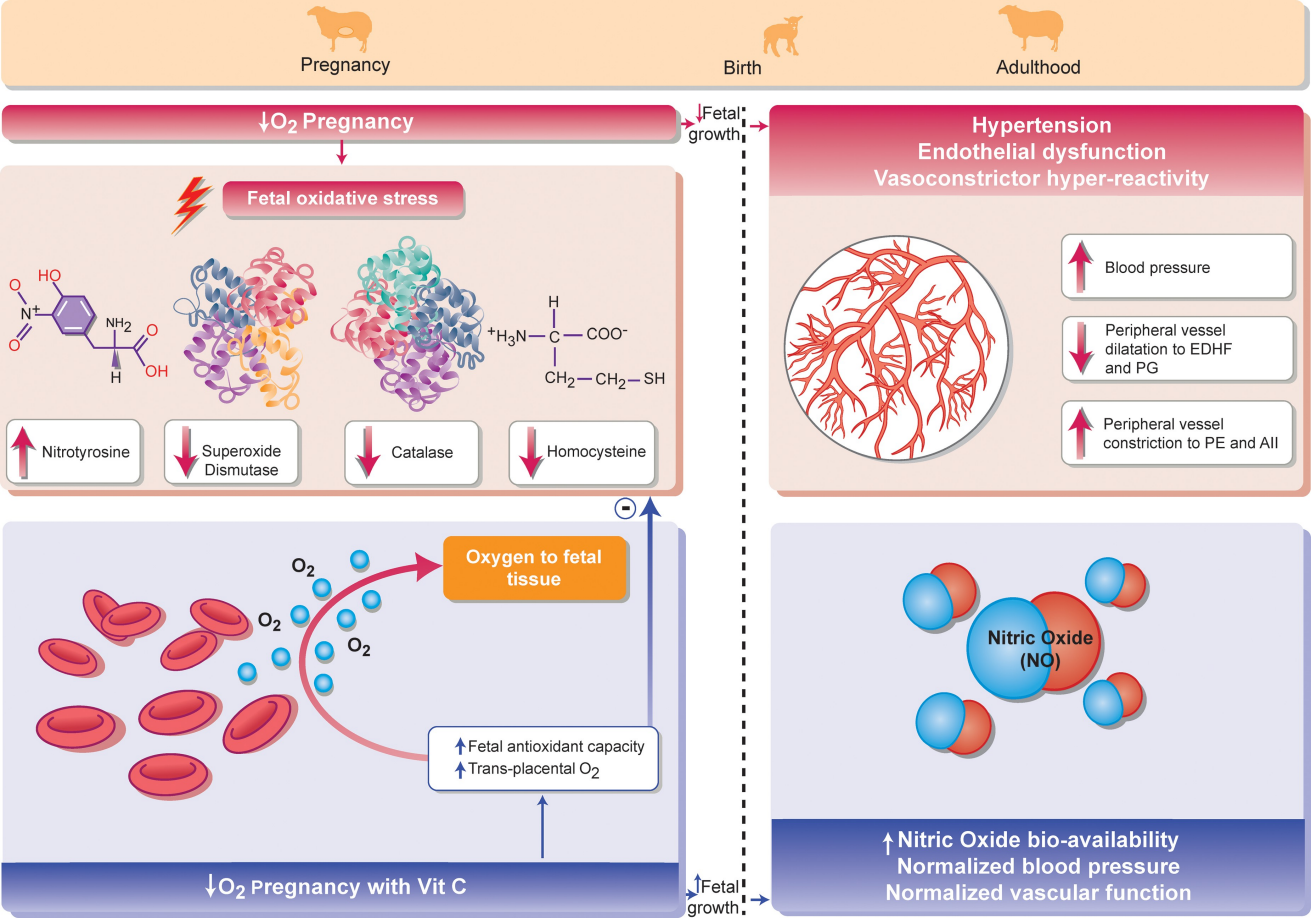
Summary illustration. Hypoxic pregnancy in sheep increases fetal oxidative stress, reduces fetal growth, and programs NO-independent endothelial dysfunction, vasoconstrictor hyper-reactivity, and hypertension in the adult offspring. Maternal treatment with vitamin C in hypoxic pregnancy increases transplacental oxygenation and fetal antioxidant capacity. This protects fetal growth and restores NO bioavailability, normalizing peripheral vascular function and arterial blood pressure in the adult offspring.

In the present study, we tested the antioxidant vitamin C, as it is widely supplemented in human populations. In an elegant study, Jackson and colleagues [37] reported that the capacity of the antioxidant vitamin C to scavenge O_2_^−^ ex vivo and its ability to prevent O_2_^−^-induced impairment of endothelial function in vivo occurred at very different concentrations, requiring a much higher effective concentration in vivo. Therefore, the dose regimen used in the preset study was derived from previous studies in our laboratory, which achieved elevations in circulating ascorbate within the required range for vitamin C to act effectively in vivo in ovine pregnancy [33,34]. To put into context, the dose of vitamin C used in the present study was 8 times higher than the dose employed in human clinical trials to prevent preeclampsia [38]. Here, we tested the hypothesis using Welsh Mountain sheep that maternal treatment with vitamin C protects against fetal growth restriction and programmed hypertension in adulthood in gestation complicated by chronic fetal hypoxia. While experimental models, which affect uterine blood flow or placental function, impair both fetal nutrition and fetal oxygenation, the isolated effect of chronic hypoxia on the fetus can be best studied by exposing the ovine pregnancy to an environment of reduced oxygenation. We therefore created 4 isobaric chambers [39,40] able to maintain pregnant sheep for long periods of gestation (Fig 1A). Adopting an integrative approach at the in vivo, isolated organ, and molecular levels, we show that maternal treatment with vitamin C in ovine hypoxic pregnancy protects against both fetal growth restriction and hypertension in the adult offspring. Mechanisms underlying this antioxidant protection include improved transplacental oxygenation, enhanced endogenous antioxidant capacity, increased in vivo NO bioavailability, offset in vivo vasoconstrictor hyper-reactivity, and replenished endothelial function in the peripheral vasculature of the offspring (see Summary illustration, Fig 2). By studying a species of similar developmental milestones to humans, the study therefore presents a conceptual advance to this field of research. It allows not only in vivo investigation of basal and stimulated cardiovascular physiology in the chronically instrumented adult offspring in an animal model that closely recapitulates human pregnancies involving fetal oxygen insufficiency, but it also provides a spring board toward human clinical translation, offering viable treatment options for the developmental origins of hypertension.

## Results

### Prenatal hypoxia ± antioxidant treatment: Effects on the mother, placenta, and fetus

In control ewes undergoing normoxic pregnancy, maternal arterial blood gases and pH remained unaltered from baseline until 135 days of gestation (dGA) (Fig 1B–1D and Table 1). However, in these ewes, there was a significant increase in maternal bicarbonate (HCO_3_^−^) on dGA 130 and dGA 135 and significant reductions in maternal hemoglobin concentration ([Hb]) from 110 dGA compared to baseline (Table 1). In control ewes, maternal plasma total levels of plasma concentrations of total NO_3_^−^ + NO_2_^−^ (NOx) also showed an increase with advancing gestation, with a significant difference from baseline at 135 dGA (Fig 1E).

**Table 1:** Maternal blood gas, acid-base, and metabolic status. Values are mean ± SEM for the maternal arterial pH, PaCO_2_, PaO_2_, Sat Hb, HCO_3_^−^, and [Hb], measured at baseline (102 until 105 dGA) and at set intervals during the experimental period (106 until 138 dGA). Groups are normoxia, hypoxia, hypoxia with vitamin C, and normoxia with vitamin C. *N* = 19 for all groups. Significant (*P* < 0.05) differences are *versus normoxia, †H versus HC, ^‡^ versus baseline, and two-way repeated-measures ANOVA with posthoc Tukey test.

		Baseline		Hypoxia/Normoxia ± Vitamin C						
		102 dGA	105 dGA	106 dGA	110 dGA	115 dGA	120 dGA	125 dGA	130 dGA	135 dGA
pH	Normoxia	7.50 ± 0.02	7.50 ± 0.01	7.51 ± 0.01	7.50 ± 0.01	7.49 ± 0.01	7.49 ± 0.01	7.49 ± 0.01	7.48 ± 0.01	7.50 ± 0.01
	Hypoxia	7.50 ± 0.01	7.50 ± 0.01	7.54 ± 0.01*‡	7.51 ± 0.01	7.51 ± 0.01	7.51 ± 0.01	7.50 ± 0.01	7.51 ± 0.01	7.50 ± 0.01
	Hypoxia with vitamin C	7.50 ± 0.01	7.52 ± 0.01	7.54 ± 0.01*‡	7.52 ± 0.01	7.52 ± 0.01	7.51 ± 0.01	7.50 ± 0.01	7.51 ± 0.01	7.51 ± 0.01
	Normoxia with vitamin C	7.49 ± 0.01	7.50 ± 0.01	7.50 ± 0.01	7.49 ± 0.01	7.49 ± 0.01	7.51 ± 0.01	7.49 ± 0.01	7.50 ± 0.01	7.49 ± 0.01
PaCO_2_ (mmHg)	Normoxia	32.5 ± 0.7	34.4 ± 0.9	35.8 ± 0.7	35.2 ± 0.8	37.0 ± 1.3	38.0 ± 1.4	36.7 ± 1.3	38.1 ± 1.5	38.1 ± 1.0
	Hypoxia	32.8 ± 0.7	33.7 ± 0.7	27.9 ± 0.6*‡	28.0 ± 0.6*‡	29.6 ± 0.8*‡	29.6 ± 0.8*‡	30.4 ± 1.7*‡	30.2 ± 0.8*‡	29.8 ± 0.9*‡
	Hypoxia with vitamin C	33 ± 1.0	33.1 ± 0.7	29.4 ± 0.8*‡	29.0 ± 0.8*‡	30.5 ± 1.2*‡	30.2 ± 0.9*‡	30.4 ± 0.8*‡	30.1 ± 0.7*‡	30.9 ± 0.8*‡
	Normoxia with vitamin C	33.3 ± 1.0	35.8 ± 0.8	36.6 ± 0.8	37.1 ± 0.8	36.1 ± 0.6	37.6 ± 0.8	37.6 ± 0.7	37.3 ± 0.9	38.1 ± 0.9
PaO_2_ (mmHg)	Normoxia	103.7 ± 5.1	101.8 ± 3.9	101.0 ± 2.3	107.3 ± 3.1	108.1 ± 1.9	105.9 ± 3.0	109.7 ± 4.8	102.1 ± 2.3	101.7 ± 2.9
	Hypoxia	108.9 ± 3.2	105.6 ± 3.0	51.7 ± 2.0*‡	48.6 ± 2.2*‡	46.2 ± 1.6*‡	47.0 ± 1.5*‡	46.4 ± 1.4*‡	46.8 ± 1.6*‡	45.2 ± 1.8*‡
	Hypoxia with vitamin C	108.2 ± 3.9	107.5 ± 3.7	51.1 ± 2.7*‡	47.9 ± 1.8*‡	45.5 ± 1.6*‡	44.8 ± 1.4*‡	44.4 ± 1.5*‡	44.7 ± 1.8*‡	44.6 ± 1.8*‡
	Normoxia with vitamin C	109.0 ± 5.0	102 ± 2.7	99.1 ± 2.1	103.4 ± 1.4	105.5 ± 3.4	101.7 ± 2.6	106.6 ± 3.0	106.0 ± 2.3	102.3 ± 2.0
Sat Hb (%)	Normoxia	102.8 ± 1.1	103.1 ± 0.5	103.0 ± 0.5	103.7 ± 0.6	103.7 ± 0.5	104.6 ± 0.5	103.9 ± 0.5	104.4 ± 0.7	103.9 ± 0.6
	Hypoxia	103.3 ± 0.4	101.4 ± 2.5	85.7 ± 2.0*‡	81.7 ± 2.2*‡	80.2 ± 2.2*‡	80.3 ± 2.4*‡	79.3 ± 3.1*‡	81.0 ± 1.9*‡	78.6 ± 2.7*‡
	Hypoxia with vitamin C	102.3 ± 1.2	103.3 ± 0.4	83.4 ± 1.9*‡	80.5 ± 2.3*‡	75.2 ± 2.3*†‡	75.4 ± 2.5*‡	73.2 ± 2.5*†‡	74.3 ± 3.1*†‡	74.0 ± 3.2*†‡
	Normoxia with vitamin C	102.9 ± 0.6	103.4 ± 0.6	102.8 ± 0.7	103.7 ± 0.3	103.6 ± 0.6	104.0 ± 0.5	104.3 ± 0.4	104.5 ± 0.4	103.9 ± 0.5
HCO_3_^−^ (mmol/L)	Normoxia	25.2 ± 1.0	26.7 ± 0.8	27.6 ± 0.6	27.0 ± 0.7	27.9 ± 0.8	28.1 ± 0.6	27.2 ± 0.7	28.5 ± 0.7‡	28.9 ± 0.6‡
	Hypoxia	25.4 ± 0.9	25.6 ± 0.7	23.6 ± 0.5*	22.3 ± 0.5*‡	23.3 ± 0.6*‡	23.3 ± 0.5*‡	23.8 ± 1.1*‡	23.6 ± 0.6*‡	23.7 ± 0.9*
	Hypoxia with vitamin C	25.7 ± 1.0	26.7 ± 0.7	24.8 ± 0.5*	23.3 ± 0.6*‡	23.9 ± 0.8*‡	23.6 ± 0.4*‡	23.5 ± 0.5*‡	24.0 ± 0.5*‡	24.0 ± 0.6*
	Normoxia with vitamin C	25.8 ± 1.0	27.2 ± 0.7	27.3 ± 0.5	27.8 ± 0.5	27.7 ± 0.7	29.8 ± 0.8‡	28.2 ± 0.7	28.4 ± 0.7‡	28.4 ± 1.0
[Hb] (g/dL)	Normoxia	11.7 ± 0.3	11.2 ± 0.3	11.0 ± 0.3	10.3 ± 0.3‡	9.5 ± 0.3‡	9.9 ± 0.4‡	10.1 ± 0.4‡	9.9 ± 0.3‡	9.7 ± 0.3‡
	Hypoxia	12.5 ± 0.3	11.8 ± 0.3	11.9 ± 0.3	11.5 ± 0.3*	11.7 ± 0.2*	11.9 ± 0.3*	12.2 ± 0.3*	12.5 ± 0.3*	12.7 ± 0.3*
	Hypoxia with vitamin C	12.2 ± 0.5	11.9 ± 0.3	12.0 ± 0.2	11.5 ± 0.3*	11.8 ± 0.2*	12.2 ± 0.2*	12.5 ± 0.2*	12.7 ± 0.2*	13.1 ± 0.3*‡
	Normoxia with vitamin C	11.1 ± 0.4	11.1 ± 0.2	10.8 ± 0.2	9.8 ± 0.3‡	9.7 ± 0.3‡	10.0 ± 0.3‡	10.1 ± 0.4‡	10.2 ± 0.4‡	10.3 ± 0.4‡

**Abbreviations:** dGA, days of gestation; [Hb], hemoglobin concentration; HCO_3_^−^, bicarbonate; PaCO_2_, arterial blood partial pressure of carbon dioxide; PaO_2_, arterial blood partial pressure of oxygen; Sat Hb, arterial blood percentage saturation of hemoglobin with oxygen.

Exposure of pregnant sheep to a 10% inspired fraction of oxygen for a month in the last third of gestation, from 105 to 135 dGA, led to a sustained controlled reduction in the maternal PaO_2_ and arterial blood percentage saturation of hemoglobin with oxygen (Sat Hb) (Fig 1B–1D). Chronic hypoxia in untreated ewes led to a transient maternal respiratory alkalosis, with significant falls in maternal arterial blood partial pressure of carbon dioxide (PaCO_2_) throughout exposure and a significant increase in maternal pH at 106 dGA, the day after the onset of hypoxia (Table 1). This maternal respiratory alkalosis was buffered by reductions in maternal HCO_3_^−^ throughout the chronic hypoxia period (Table 1). In contrast to control ewes undergoing normoxic pregnancy, ewes exposed to chronic hypoxia did not show a significant fall from baseline in maternal [Hb] and levels of maternal [Hb] were significantly higher than those in control ewes from 110 dGA (Table 1). Maternal hypoxia in untreated ewes enhanced the ontogenic increase in maternal plasma NOx (Fig 1E), and it did not affect maternal food intake or maternal weight gain (Fig 1F and 1G).

Maternal vitamin C treatment in both control and hypoxic ewes produced similar increments from baseline (N: 38.0 ± 3.9; H: 36.2 ± 3.1 μmol/L) in maternal plasma vitamin C, doubling the circulating concentration (Fig 1H). Maternal vitamin C treatment in hypoxic pregnancy did not affect the alterations in maternal blood gases or pH or maternal plasma NOx seen in untreated hypoxic ewes. In addition, ewes undergoing hypoxia treated with vitamin C also did not show any changes in maternal food intake (Fig 1B–1F and Table 1). However, maternal treatment with vitamin C in hypoxic pregnancy led to a greater fall in maternal Sat Hb despite a similar fall in maternal PaO_2_ compared to untreated ewes (Fig 1C and 1D)_._ This meant a rightward shift in the oxygen–hemoglobin dissociation curve and thereby a significant increase in the maternal arterial P_50_ from 23.5 ± 0.9 to 26.6 ± 10 mmHg (*P* < 0.05; Fig 1I). The total number of placentomes, the placental weight, the fetal:placental weight ratio, and the distribution of placentome type did not differ among all 4 groups (see S1A–S1C Fig).

**Fig 3:**
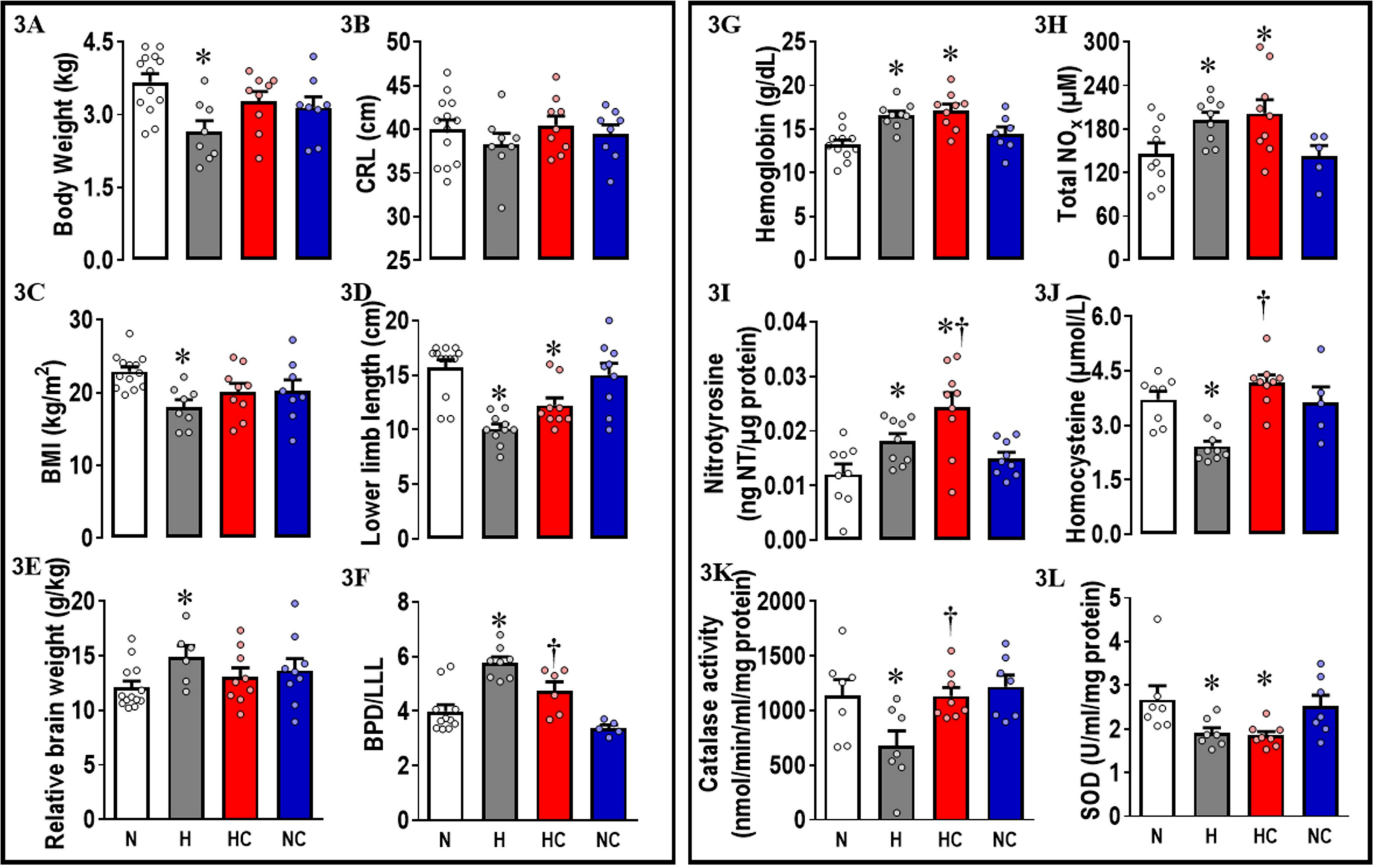
Fetal offspring data. Measured at 138 dGA: 3A, fetal body weight; 3B, CRL; 3C, BMI; 3D, LLL; 3E, brain weight relative to body weight; 3F, BPD to LLL ratio; 3G, blood concentration of hemoglobin; 3H, NOx; 3I, liver concentrations of 3-nitrotyrosine; 3J, plasma concentrations of homocysteine; 3K, liver catalase activity; and 3L, liver SOD activity. Values are mean ± SEM for all data. Groups are N (open symbols, *n* = 7–13), H (grey symbols, *n* = 6–10), HC (red symbols, *n* = 6–9), and NC (blue symbols, *n* = 5–9). Significant (P < 0.05) differences are the following: *versus normoxia, †H versus HC, and two-way ANOVA with posthoc Tukey test. BMI, body mass index; BPD, biparietal diameter; CRL, crown rump length; dGA, days of gestation; H, hypoxia; HC, hypoxia with vitamin C; LLL, lower limb length; N, normoxia; NC, normoxia with vitamin C; NOx, plasma concentrations of total NO_3_^−^ + NO_2_^−^; SOD, superoxide dismutase.

Fetuses from hypoxic pregnancy showed growth restriction with brain sparing. They had a significant reduction in body weight, body mass index (BMI), and lower limb length (LLL) and significant increases in the absolute and relative brain weight, in the relative weight of the hypothalamus, the absolute weight of the cerebral hemispheres, and in the ratio of the biparietal diameter relative to LLL when compared to fetuses from normoxic pregnancy (Table 2 and Fig 3A–3F). Fetuses from hypoxic pregnancy showed an increase in [Hb] and in plasma total NOx (Fig 3G and 3H). They also had increased hepatic nitrotyrosine concentrations and decreases in plasma homocysteine and in the activities of catalase and superoxide dismutase (SOD) in the liver (Fig 3I–3L).

**Table 2:** Fetal and adult offspring organ weights. Values are mean ± SEM for the fetal offspring organ weights at 138 dGA and adult offspring organ weights at PM. Groups are normoxia (fetus, *n* = 12; adult, *n* = 9), hypoxia (fetus, *n* = 6; adult, *n* = 9), hypoxia with vitamin C (fetus and adult, *n* = 9), and normoxia with vitamin C (fetus and adult, *n* = 9). Significant (*P* < 0.05) differences are *versus normoxia, †hypoxia versus hypoxia with vitamin C, and two-way ANOVA with posthoc Tukey test.

	Normoxia	Hypoxia	Hypoxia with vitamin C	Normoxia with vitamin C
**Fetal offspring**	** **	** **	** **	** **
***Brain***				
Absolute (g)	43.3 ± 0.7	38.9 ± 2.3*	41.5 ± 1.0	44.0 ± 1.5
Relative (g/kg)	12.1 ± 0.6	14.9 ± 1.0*	13.0 ± 0.8	13.7 ± 1.1
*Hypothalamus*				
Absolute (g)	3.7 ± 0.2	4.08 ± 0.2	4.3 ± 0.1	4.2 ± 0.2
Relative to brain	0.086 ± 0.006	0.106 ± 0.004*	0.105 ± 0.004*	0.096 ± 0.004
*Cerebellum*				
Absolute (g)	4.6 ± 0.2	4.0 ± 0.2	4.5 ± 0.1	4.6 ± 0.2
Relative to brain	0.106 ± 0.003	0.104 ± 0.002	0.108 ± 0.003	0.105 ± 0.002
*Hemispheres*				
Absolute (g)	33.6 ± 0.9	28.5 ± 1.8*	29.8 ± 1.5	32.7 ± 1.5
Relative to brain	0.780 ± 0.016	0.733 ± 0.007	0.717 ± 0.030	0.756 ± 0.010
***Heart***				
Absolute (g)	33.6 ± 2.5	26.3 ± 3.0	32.2 ± 1.6	33.7 ± 2.2
Relative (g/kg)	9.2 ± 0.6	9.7 ± 0.2	10.0 ± 0.5	10.3 ± 0.7
***Liver***				
Absolute (g)	78.8 ± 5.2	56.6 ± 7.2	69.5 ± 5.8	67.2 ± 8.4
Relative (g/kg)	21.4 ± 0.8	21.0 ± 1.8	21.1 ± 1.2	18.8 ± 1.0
**Adult offspring**	** **	** **	** **	** **
***Brain***				
Absolute (g)	79.9 ± 1.8	84.6 ± 2.1	82.0 ± 2.1	79.8 ± 2.5
Relative (g/kg)	3.2 ± 0.2	2.8 ± 0.1	3.0 ± 0.2	2.8 ± 0.1
*Hypothalamus*				
Absolute (g)	8.6 ± 0.3	9.2 ± 0.3	9.2 ± 0.3	8.6 ± 0.5
Relative to brain	0.108 ± 0.003	0.109 ± 0.002	0.112 ± 0.004	0.107 ± 0.004
*Cerebellum*				
Absolute (g)	10.4 ± 0.3	10.6 ± 0.3	10.2 ± 0.4	10.1 ± 0.4
Relative to brain	0.130 ± 0.003	0.125 ± 0.002	0.124 ± 0.004	0.127 ± 0.002
*Hemispheres*				
Absolute (g)	60.9 ± 1.4	64.8 ± 1.6	62.7 ± 1.7	61.1 ± 1.7
Relative to brain	0.762 ± 0.004	0.766 ± 0.003	0.764 ± 0.005	0.766 ± 0.005
***Heart***				
Absolute (g)	221.9 ± 10.9	239.5 ± 10.7	239.4 ± 9.1	226.4 ± 8.5
Relative (g/kg)	8.4 ± 0.3	8.0 ± 0.4	8.6 ± 0.5	8.0 ± 0.3
***Liver***				
Absolute (g)	397.4 ± 18.9	441.9 ± 29.0	407.6 ± 11.5	457.8 ± 16.8
Relative (g/kg)	15.3 ± 1.2	14.9 ± 1.2	14.7 ± 0.9	16.2 ± 0.8

Fetuses from hypoxic pregnancy treated with maternal vitamin C no longer showed significant reductions in body weight and BMI, no longer showed a significant increase in relative brain weight, and the ratio of the biparietal diameter relative to LLL was restored when compared to fetuses from normoxic pregnancy. However, the reduction in LLL and the increase in the relative weight of the hypothalamus persisted (Table 2 and Fig 3A–3F). Fetuses from hypoxic pregnancy treated with maternal vitamin C still showed an increase in [Hb] and total plasma NOx and a fall in liver SOD, and the increase in hepatic nitrotyrosine was significantly greater than in fetuses from untreated hypoxic pregnancy. However, they showed restored levels of plasma homocysteine and of hepatic catalase activity when compared to hypoxic fetuses of untreated pregnancy (Table 2 and Fig 3J and 3K).

Maternal treatment with vitamin C in normoxic pregnancy had no effect on any outcome variable measured in the ewes or fetuses when compared to untreated normoxic pregnancy (Tables 1 and 2, Figs 1 and 3).

When blood was taken at post mortem immediately following the end of the experiment at 138 dGA, the fetal plasma concentrations of cortisol were not different among all groups (N: 17.6 ± 3.0; H: 16.6 ± 2.9; HC: 27.7 ± 2.9; NC: 28.5 ± 6.2 ng.mL^−1^). We have also reported that maternal plasma stress hormone mean levels are not different in ewes undergoing normoxic or chronic hypoxic pregnancy [39].

### Prenatal hypoxia ± antioxidant treatment: Effects on the adult offspring

A separate cohort of animals was allowed to deliver naturally at term under normoxic conditions, and lambs were maintained with their mothers until weaning and then lived on pasture in fields surrounding the Barcroft Centre at the University of Cambridge. Lambs from hypoxic pregnancy were born earlier in gestation (N: 147.4 ± 0.6 versus H: 143.6 ± 0.6 days, *P* < 0.05) with a reduced birth weight (N: 3.5 ± 0.1 versus H: 2.9 ± 0.1 Kg, *P* < 0.05). Despite being born lighter, lambs from hypoxic pregnancy showed accelerated postnatal fractional growth rates (N: 25.1 ± 1.2 versus H: 32.4 ± 1.8 mg.d^−1^, starting weight^−1^, *P* < 0.05), such that body weight at 9 months was no longer different (N: 26.5 ± 1.2 versus H: 30.4 ± 1.7 Kg). There were also no differences in the weight of the brain, heart, and liver nor in the weight of components of the brain, such as the hemispheres, hypothalamus, or cerebellum between lambs from normoxic or hypoxic pregnancy at 9 months (Table 2).

**Fig 4:**
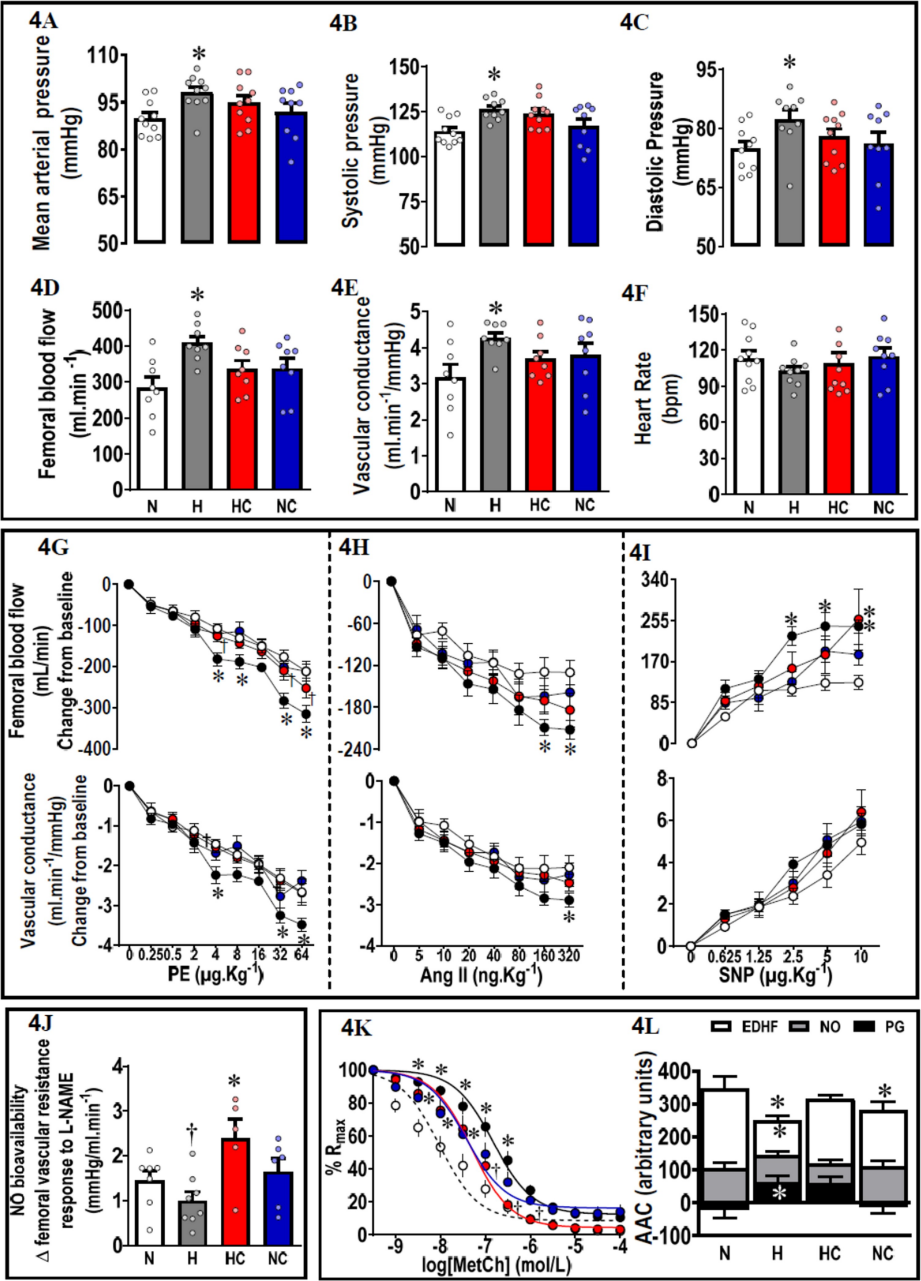
Adult offspring in vivo and in vitro cardiovascular function. Adult offspring in vivo basal: 4A, mean arterial pressure; 4B, systolic pressure; 4C, diastolic pressure; 4D, femoral blood flow; 4E, femoral vascular conductance; and 4F, heart rate. Adult offspring in vivo stimulated femoral blood flow and femoral vascular conductance responses to 4G, PE; 4H, Ang II; and 4I, SNP. 4J. Femoral vascular resistance response after 10 minutes of L-NAME exposure (change from baseline). 4K. In vitro concentration–response curves of the percent Rmax to methacholine. 4L. The NO-dependent, EDHF-dependent, and PG-dependent components (AAC) of vasorelaxation in second-order femoral resistance arteries isolated from 9-month-old adult offspring. Values are mean ± SEM for all data. Groups are N (open symbols, *n* = 6–11), H (black/grey symbols, *n* = 6–11), HC (red symbols, *n* = 5–11), and NC (blue symbols, *n* = 6–15). Significant (*P* < 0.05) differences are *versus normoxia, †H versus HC, one- or two-way ANOVA with posthoc Tukey test (4A–4F, 4J and 4L), or two-way repeated measures ANOVA with posthoc Tukey test (4G–4I, 4K). AngII, angiotensin II; AAC, area above the curve; EDHF, endothelium derived hyperpolarising factor; H, hypoxia; HC, hypoxia with vitamin C; L-NAME, N(ω)-nitro-L-arginine methyl ester; N, normoxia; NC, normoxia with vitamin C; PE, phenylephrine; percent Rmax, maximal femoral vessel relaxation; PG, prostaglandin; SNP, sodium nitroprusside.

In vivo experiments in chronically instrumented 9-month-old lambs revealed that offspring from hypoxic pregnancy were hypertensive, and they showed greater resting values for femoral blood flow and vascular conductance (Fig 4A–4F). Addressing in vivo mechanisms contributing to the hypertension, adult offspring of hypoxic pregnancy showed enhanced in vivo femoral constrictor responses to phenylephrine (PE) and to angiotensin II (AngII). They also showed enhanced in vivo femoral dilator responses to sodium nitroprusside (SNP), with no significant effect on basal femoral vascular resistance of N(ω)-nitro-L-arginine methyl ester (L-NAME) treatment (Fig 4G–4J). To further address mechanisms, third order femoral vessels isolated from adult offspring of hypoxic pregnancy showed reduced relaxation to methacholine because of endothelial dysfunction due to a decrease in NO-independent mechanisms, including prostanoid and endothelium-derived hyperpolarizing factor (EDHF) dilator reactivity (Fig 4K and 4L).

Adult offspring of hypoxic pregnancy treated with maternal vitamin C were no longer hypertensive (Fig 4A–4C); they showed a significant increase in femoral vascular resistance following L-NAME treatment, indicating greater circulating NO bioavailability (Fig 4J), and had constrictor and dilator responses in vivo and ex vivo, which were more similar to adult offspring of control pregnancy (Fig 4G–4L). The weight of organs collected at 9 months was similar in adult offspring from treated and untreated hypoxic pregnancies (Table 2).

Adult offspring of normoxic pregnancy treated with vitamin C showed similar responses in all outcomes relative to offspring of untreated normoxic pregnancy, with the exception of reduced endothelial function in isolated femoral vessels (Table 2 and Fig 4L).

## Discussion

Any pregnancy in which there is an increase in placental vascular resistance will impact oxygen delivery to the fetus. Therefore, chronic fetal hypoxia is one of the most common complications of human pregnancy and is known to cause fetal growth restriction [13,14]. Recently, we created isobaric chambers able to maintain pregnant sheep for long periods of gestation under highly controlled hypoxic conditions [39,40]. We have used this novel technology to model the short- and longer-term effects of isolated hypoxic pregnancy on the cardiovascular system of the offspring in a species of similar temporal developmental milestones to humans.

### Hypoxic pregnancy, fetal growth, and fetal brain sparing

During healthy pregnancy, maternal cardiovascular adaptations are essential to maintain appropriate fetal growth and development as well as the maternal well-being. These adaptations include the expansion of the maternal blood volume, which places the mother in a state of physiological anemia. Following Poiseuille’s Law, reductions in maternal [Hb] decrease the maternal blood viscosity, thereby promoting lower systemic vascular resistance and increased blood flow in the uteroplacental circulation to match fetal growth in the last third of pregnancy [41,42]. Therefore, the fall in maternal [Hb] in control ewes in the present study is consistent with healthy maternal adaptations during late gestation. In humans and sheep, exposure to hypoxia during pregnancy, such as that associated with high altitude, leads to a fall in the maternal PaO_*2*_ and PaCO_*2*_ and an increase in maternal [Hb] [42–44]. In human high-altitude pregnancy, there is also an increase in the maternal blood pH and a decrease in HCO_*3*_^*−*^ aiming to buffer the maternal alkalosis [43,44]. Therefore, the maternal circulatory responses to chronic hypoxia in the present study are consistent with these maternal adaptations to hypoxic pregnancy, and they explain the significantly higher levels of maternal [Hb] and lack of a fall in maternal [Hb] with advancing gestation in the chronically hypoxic relative to the normoxic ewe.

Fetuses of hypoxic pregnancy were not only lighter but also showed a reduced BMI with an increase in relative brain weight. These are robust indices of fetal brain sparing at the expense of redistribution of blood flow away from the fetal peripheral circulation [14]. Regional differences in brain weight changes suggest that the hypothalamus and cerebellum were preserved more so than the hemispheres. Seminal studies in fetal sheep have previously reported a hierarchy in maintaining blood flow to subcortical regions at the expense of blood flow to the brain hemispheres during chronic hypoxia [45]. In a separate study, we have previously reported that a comparative level of maternal hypoxia in sheep, as used in the present study, reduced the fetal PaO_*2*_ from normal baseline to values of 12 *±* 1 mmHg [40]. This is of significant clinical translation, as this is the level of reduced fetal oxygenation measured by cordocentesis in human pregnancies complicated by significant fetal growth restriction [46].

### Hypoxic pregnancy and oxidative stress in the fetal cardiovascular system

Additional data in the present study show that increased oxidative stress in the fetus contributes to the mechanisms mediating the adverse consequences of developmental hypoxia. 3-nitrotyrosine (3-NT) is a product of protein tyrosine nitration following oxidative damage to proteins by peroxynitrie [47]. An increase in oxidative stress will cause an increased demand for the synthesis of endogenous antioxidant enzymes. Therefore, a decrease in the activities of hepatic catalase and SOD in fetuses from hypoxic pregnancy provides further evidence of increased oxidative stress and impaired antioxidant defenses in the hypoxic fetus in the present study.

It is widely recognized that individuals with high-plasma homocysteine (Hcy) levels have an increased risk of developing cardiovascular disease [48]. However, it must also be recognized that Hcy is an important intermediate in the conversion of methionine to cysteine, which is then used in the production of the antioxidant glutathione. During an oxidative challenge, this pathway is up-regulated rapidly [48,49]. However, in situations of low Hcy, there is a limit to how much glutathione can be produced, thereby restricting the body’s ability to respond to oxidative stress [49]. Any condition that causes an increase in oxidative stress will promote an increased demand on the liver to produce glutathione, and consequently, low availability of glutathione and homocysteine is also associated with a large range of diseases [49]. In hypoxic pregnancies, oxidative stress will drive Hcy into glutathione synthesis, leading to a fall in plasma Hcy levels, as was observed with the hypoxic fetuses in the present study. These data in the sheep fetus are also in keeping with reports of oxidative stress in the fetal cardiovascular system in rodent models of hypoxic pregnancy [8,28–31,35,36].

In the present study, treatment of hypoxic pregnancies with vitamin C maintained the increase in fetal [Hb], and in fetal total plasma NOx, it augmented the increase in nitrotyrosine in the fetal liver but prevented the fall in the fetal hepatic activities of Hcy and catalase without affecting the reduction in SOD in the fetus of hypoxic relative to normoxic pregnancy. Maintained or augmented increases in fetal [Hb], fetal total plasma NOx, and in nitrotyrosine in the fetal liver in hypoxic pregnancy suggests that maternal treatment with vitamin C is not protective in hypoxic pregnancy by affecting oxygen sensing pathways, the induction of oxidative stress, or compensatory adaptations to increase NO bioavailability in the fetus. Rather, maternal treatment with vitamin C in hypoxic pregnancy confers antioxidant protection by additional exogenous antioxidant supplementation. This normalizes the fetal hepatic activities of Hcy and catalase but not SOD, thereby reducing the demand for 2 out of 3 fetal endogenous antioxidant defenses in hypoxic pregnancy.

### Maternal vitamin C protects fetal growth and prevents programmed systemic hypertension in the adult offspring in hypoxic pregnancy

Maternal vitamin C treatment increases umbilical blood flow in vivo in ovine pregnancy by quenching O_*2*_^*−*^ production and increasing NO bioavailability [33,34]. Here, we show that maternal vitamin C treatment additionally increases the maternal P_*50*_ and restores fetal plasma Hcy levels and hepatic catalase activity. Therefore, maternal vitamin C treatment by increasing umbilical blood flow, fetal endogenous antioxidant defenses, and the transplacental PO_*2*_ gradient will buffer reductions in fetal oxygen delivery despite chronic hypoxia. These mechanisms may explain the capacity of the chronically hypoxic fetus whose mother is treated with vitamin C to be able to maintain appropriate growth, no longer requiring fetal brain sparing. Pregnancy complicated by fetal growth restriction is itself a major killer in perinatal medicine today [15]. Therefore, treatment to protect fetal growth in human high-risk pregnancy is clinically important.

Systemic hypertension affects 1 billion adults worldwide [50]. Increased systolic blood pressure is an independent risk factor for coronary events, stroke, and heart failure [51]. A few studies in rodents have shown that hypoxic pregnancy can program hypertension in the adult offspring and that this was associated with constrictor hyper-reactivity in the peripheral vasculature and exacerbated cardiovascular responses to stress [52–55]. However, intervention against the programmed hypertension was not addressed in these studies. Here, we show that ovine pregnancy complicated by developmental hypoxia can also program significant hypertension in the adult offspring. The mechanisms mediating hypertension in the present study include increased in vivo vasoconstrictor reactivity to α-adrenergic agonists and to AngII in the peripheral circulation. In addition, we show endothelial dysfunction due to impaired EDHF and prostanoid-dependent reactivity in femoral arteries of adult offspring of hypoxic pregnancy. There is also in vivo evidence of programmed compensatory increases in NO function in the cardiovascular system in adult offspring of hypoxic pregnancy. We show in vivo that they have enhanced basal femoral blood flow and vascular conductance and a greater femoral dilator response to SNP, a NO donor. In the present study, maternal vitamin C treatment in hypoxic pregnancy prevented hypertension in the adult offspring. Therefore, we provide novel in vivo evidence of successful intervention with antenatal maternal antioxidant administration against programmed systemic hypertension in the adult offspring. Further, the successful intervention against programmed hypertension is in an ovine model of chronic fetal hypoxia, thereby of comparable developmental milestones to humans. In the present study, maternal treatment with vitamin C conferred protection against hypertension in the offspring by programming an increase in NO bioavailability, since in vivo treatment of adult offspring with L-NAME led to significantly greater increases in femoral vascular resistance. Further, maternal vitamin C also improved endothelial function. Combined, therefore, these protective mechanisms induced by maternal vitamin C treatment meant that NO-compensatory responses in the peripheral vasculature of adult offspring of hypoxic pregnancy, such as enhanced basal femoral blood flow and vascular conductance and a greater femoral dilator response to SNP, were no longer required.

In the present study, fetal sheep were not instrumented with catheters, as the focus was on offspring of hypoxic pregnancy being allowed to be born naturally and maintained until adulthood for cardiovascular study. This study design precluded the measurement of fetal arterial blood pressure and knowledge of whether chronic fetal hypoxia led to fetal arterial hypertension in utero, which was maintained or exacerbated until adulthood. However, in separate studies, which determined changes in fetal arterial blood pressure during hypoxic pregnancy in sheep using isobaric chambers, we [40] and others [56] have reported that the chronically hypoxic fetus showed an impaired or unchanged ontogenic increase in basal arterial blood pressure. Similarly, late-gestation fetal sheep conceived, gestated, and studied at high altitude had lower resting arterial blood pressure relative to sea level controls [57]. Combined, therefore, past and present evidence suggests that chronic fetal hypoxia programs adult-onset hypertension and that this develops in association with aging, rather than it persisting from fetal origin.

Several studies in rodents and in sheep have reported that pregnancy exposed to excess glucocorticoids can also program hypertension in the adult offspring [58–62]. In the present study, fetal plasma glucocorticoid levels were not elevated by the end of hypoxic pregnancy relative to normoxic pregnancy. This is again consistent with data derived from high-altitude pregnancy, which reports in sheep that the fetal hypothalamo–pituitary–adrenal axis desensitizes in response to chronic hypoxia. This is an adaptive response to prevent stress-induced preterm birth and to maintain normal circulating levels of fetal cortisol for appropriate development and maturation [63]. Therefore, in the present study, chronic fetal hypoxia programmed adult-onset hypertension independent of fetal hypercostisolemia.

In the clinical setting, people are diagnosed as hypertensive when the systolic/diastolic reading exceeds 140/90, with systolic blood pressure being the greatest predictor of later disease (National Institute for Health and Care Excellence [NICE] guidelines) [64]. There is no equivalent definition for sheep, and although a direct comparison cannot be made, adult offspring of hypoxic pregnancy in the present study had a mean increase in systolic and diastolic blood pressure values of 13 and of 8 mmHg above control values, respectively. The magnitude of these effects is of high clinical significance, as reports on the prevention and treatment of high blood pressure in humans show that the risk of cardiovascular disease doubles with each increment of systolic/diastolic pressure of 20/10 mmHg and that such individuals require active health-promoting lifestyle modifications to prevent overt cardiovascular disease [18]. The Framingham study reported that modest increases in mean blood pressure milder than those observed in lambs from hypoxic pregnancy in the present study can dramatically increase the risk of a future cardiovascular event [65]. A recent randomized clinical trial also reported that targeting a systolic blood pressure to <120 mmHg compared with <140 mmHg in humans resulted in lower rates of fatal and nonfatal cardiovascular events [66]. In the present study, offspring of hypoxic pregnancy therefore already fall under the human classification of prehypertensive despite being young adult and female and thereby likely to show even greater risk of cardiovascular disease with aging.

Interestingly, adult offspring born from normoxic pregnancy treated with vitamin C also showed evidence of endothelial dysfunction in the present study. While antioxidant supplementation may benefit conditions of increased oxidative stress, antioxidant excess resulting from antioxidant supplementation under normal conditions may paradoxically promote oxidative stress [32]. One mechanism may be by providing excess NO bioavailability, which also serves as a precursor for peroxynitrite generation [32]. This reveals an equally important translational message in the present study, and that is that clinically, maternal treatment with antioxidants should only be administered to pregnancies diagnosed with chronic fetal hypoxia rather than given prophylactically to all pregnancies. This will only be feasible if risk can be identified prior to maternal therapy. Expert obstetric opinion today dictates that in human pregnancy, chronic fetal hypoxia can be reliably identified according to severity by ultrasound scan between 20–26 weeks of gestation. Diagnosis encompasses early onset intrauterine growth restriction, reduced fetal heart rate variability and body movements and/or abnormal Doppler blood flow velocimetry indices in the fetal middle cerebral artery and the umbilical circulation [67,68]. Although this supports possible translation to the human clinical setting, there are several important points for additional discussion and further key basic science experiments that are required to inform double-blind, randomized, placebo-controlled human clinical trials before maternal antioxidant supplementation in human obstetric practice could be considered.

### Limitations

A limitation of the current program of work is that although the study design controlled for sex differences in the offspring, it did not address them. To make the study viable ethically and economically, every singleton pregnancy generated was used. Therefore, studies in the fetal period used the male offspring while studies in the adult period used the female offspring, as ewe lambs are easier to group house when compared to growing rams. Longitudinal comparisons from the fetal through the adult period thus require caution.

A second limitation of the current program of work is that outcome variables in the fetal and adult periods were not balanced for sex. Several recent reviews have reported important sex-dependent differences in the programming effects on cardiovascular function in the adult offspring of adverse intrauterine conditions, usually highlighting that the male offspring is more vulnerable [69–71]. It is also well accepted that aging increases cardiovascular risk [72–74] and that ovarian estrogens at adulthood confer protection against cardiovascular disease [75]. Given that the present study reported significant hypertension in young adult females born from hypoxic pregnancy, the data may underestimate the potential adverse impact of developmental hypoxia on systemic hypertension and cardiovascular dysfunction in adult male offspring in this ovine model of hypoxic pregnancy. However, the study does emphasize that hypoxic pregnancy in sheep increases the risk of cardiovascular dysfunction and that maternal antioxidant treatment is protective in both male and female offspring.

A third limitation of the current study design is that pregnancies exposed to hypoxia from 105 to 135 dGA were returned to normoxia at 135 days to allow delivery under normal oxygen conditions. This study design ensured a fixed length period of fetal exposure to chronic hypoxia prior to birth. However, it also means that adult-onset hypertension could be programmed by fetal exposure to chronic hypoxia with some reoxygenation rather than by chronic fetal hypoxia alone. Since hypoxia and reoxygenation is a more potent stimulus of oxidative stress than hypoxia alone [32], the data highlight the protective impact of maternal antioxidant therapy even under these conditions.

The final limitation and perhaps the most important in terms of human clinical translation is that multicenter human clinical studies have been unable to confirm benefits of maternal treatment with antioxidant vitamins in pregnancies at risk of preeclampsia [38,76]. These studies include the vitamins in preeclampsia (VIP) and international trial of antioxidants in the prevention of preeclampsia (INTAPP) trials, which found that administration of vitamin C (1 g.d^*−1*^) and E (400 IU) to mothers once clinical signs of the preeclampsia were established did not affect the incidence of preeclampsia but instead led to an increase in the rate of low-birth weight babies by 4%. While preeclampsia was not the focus of the present study and the adverse effect on birth weight of maternal vitamin C in human studies appears no longer present with meta-analysis [38,76], these clinical findings are significant enough to warn against possible adverse side effects of maternal vitamin C treatment in human pregnancy. Hence, we strongly agree that vitamin C may not be the antioxidant of choice for translation to human therapy. However, the data we show provide proof-of-principle that maternal treatment with antioxidants can protect against developmental programming of adult-onset hypertension. Future key experiments to inform human clinical trials should consider alternative maternal antioxidant therapy of improved translational value using human clinically relevant animal models of adverse pregnancy, such as sheep. Plausible alternative candidate antioxidant therapies for human translation may include melatonin, allopurinol, or the mitochondria-targeted antioxidant MitoQ. Rodent models show that maternal treatment with melatonin confers protection on fetal growth and cardiovascular function in the offspring of adverse pregnancy at doses similar to or lower than those required to avoid jet lag in humans [77,78]. An alternative antioxidant strategy may be to prevent the synthesis of free radicals through specific pathways rather than quench them once formed, as in the case of the xanthine oxidase inhibitor allopurinol. Hypoxia is a potent stimulus for xanthine oxidase-induced superoxide anion generation [32], and recently, we have reported that maternal treatment with allopurinol in hypoxic pregnancy in rats protects against cardiac dysfunction in the adult offspring [79]. Yet another strategy may be for targeted antioxidant therapy. Mitochondria are a major site of ROS production; therefore, targeting them specifically should be one of the most effective antioxidant therapeutic strategies. This is now possible with mitochondria-targeted antioxidants. MitoQ is composed of a lipophilic triphenylphosphonium cation covalently attached to an ubiquinol antioxidant [80,81]. Lipophilic cations can easily move through phospholipid bilayers without requiring a specific uptake mechanism. Therefore, the triphenylphosphonium cation concentrates MitoQ several hundred-fold within the mitochondria, driven by the large mitochondrial membrane potential [78,79]. Only within the mitochondria, MitoQ is reduced by the respiratory chain to its active ubiquinol form, which is a particularly effective antioxidant that prevents lipid peroxidation and mitochondrial damage [80,81]. The benefits of MitoQ have been revealed in a range of in vivo studies in rats and mice and in two human trials [82–85]. In contrast to vitamin C and other conventional antioxidants, MitoQ demonstrates no pro-oxidant activity at high doses and long-term administration to mice for 28 weeks [83] and to human patients in two Phase II trials, one that lasted 1 year revealed no toxicity [84,85]. Two recent studies in rats have also reported that nano-particle bound MitoQ, which prevents passage of the drug through the placenta and to the fetus, protected against programmed cardiac diastolic dysfunction, endothelial dysfunction, and neurodegeneration in the adult offspring, using an established model of hypoxic pregnancy in rats [35,86]. However, the antioxidant benefits of either melatonin or allopurinol or MitoQ in protecting against fetal growth restriction and programmed hypertension at adulthood in offspring of high-risk pregnancy in sheep have yet to be determined.

In conclusion, our discoveries provide compelling evidence of clinical translational importance for chronic fetal hypoxia programming of adult-onset hypertension in a species of similar developmental milestones to humans. Further, the data provide novel insight into underlying mechanisms and thereby successful intervention against cardiovascular dysfunction in the next generation programmed by pregnancy complicated by developmental hypoxia (See Summary illustration, Fig 2).

## Materials and methods

### Ethics statement

All procedures were performed under the Home Office Project Licence PC6CEFE59 under the Animals (Scientific Procedures) Act 1986 Amendment Regulations 2012, following ethical review by the University of Cambridge Animal Welfare and Ethical Review Board (AWERB).

### Surgical preparation of pregnant ewes

At 100 ± 1 days gestational age (term ca. 145 days), pregnant Welsh Mountain ewes carrying singleton pregnancies determined by ultrasound scan (Toshiba Medical Systems Europe, Zoetermeer, the Netherlands) underwent a laparotomy and catheterization, as previously described [33,34,39,40]. In brief, under general anesthesia (1.5%–2.0% isofluorane in 60:40 O_2_:N_2_O) maintained by use of a positive pressure ventilator (Datex-Ohmeda Ltd, Hatfield, Hertfordshire, UK), a midline abdominal incision and uterotomy was used to expose the fetal hind limbs and determine fetal sex. If male, then the fetuses were assigned to the fetal studies group; female fetuses were assigned to the adult offspring studies group. The fetus was returned into the intrauterine cavity, and the uterine and maternal abdominal incisions were closed in layers. A Teflon catheter (i.d. 1.0 mm, o.d. 1.6 mm, Altec, UK) was placed in the maternal femoral artery and positioned in the descending aorta, in addition to a maternal femoral venous catheter into the inferior vena cava (Critchly Electrical Products, NSW, Australia). Catheters were filled with heparinized saline (80 i.u.ml^−1^ heparin in 0.9% NaCl), tunnelled subcutaneously, and exteriorized via a keyhole incision made in the maternal flank to be kept inside a plastic pouch sewn onto the maternal skin.

Following surgery, ewes were housed in individual floor pens with a 12:12-hour light–dark cycle. From 103 dGA, ewes were fed daily a bespoke maintenance diet made up of concentrate and hay pellets to facilitate the monitoring of food intake (Cambridge ewe diet: 40g nuts/kg and 3g hay/kg; Manor Farm Feeds Ltd; Oakham, Leicestershire, UK) [39,40]. On day 105 of gestation, ewes were randomly assigned to 1 of 4 experimental groups: normoxia (N), chronic hypoxia (H), chronic hypoxia with vitamin C treatment (HC), and normoxia with vitamin C (NC); *n* = 18 for all groups.

### Chronic hypoxia protocol

Ewes were housed in bespoke isobaric hypoxic chambers (Telstar Ace, Dewsbury, West Yorkshire, UK) supplied with controlled volumes of nitrogen and air provided by nitrogen generators and air compressors, respectively, from a specially designed nitrogen generating system (Domnick Hunter Gas Generation, Gateshead, Tyne & Wear, UK) [39,40]. Ambient PO_2_, PCO_2_, humidity, and temperature within each chamber were monitored via sensors, displayed, and values recorded continuously via the Trends Building Management System of the University of Cambridge through a secure Redcare intranet. In this way, the percentage of oxygen in the isolators could be controlled with precision over long periods of time. For experimental procedures, each chamber had a double transfer port to internalize material and a manually operated sliding panel to bring the ewe into a position in which daily sampling of blood could be achieved through glove compartments. Each chamber incorporated a drinking bowl on continuous water supply and a rotating food compartment, which could be removed for determining food intake. Therefore, all experimental and maintenance procedures could be carried out without interruption of the hypoxic exposure. Pregnancies randomly assigned to the chronic hypoxia group were placed inside the chambers at 103 dGA under normoxic conditions (11 L.sec^−1^ air, equating to 39.6 m^3^.h^−1^). At 105 days, pregnancies were exposed to ca. 10% O_2_ by altering the inspirate mixture to 5 L.sec^−1^ air: 6 L.sec^−1^ N_2_. The inspirate air mixture underwent a minimum of 12 changes per hour in each chamber, and the incoming air mixture was passed via silencers able to reduce noise levels within the hypoxic chamber laboratory (76 dB[A]) and inside each chamber (63 dB[A]) to values lower than those necessary to abide by the Control of Noise at Work Regulations. This not only complied with human health and safety and animal welfare regulations but also provided a highly tranquil environment for the animal inside each chamber.

### Maternal vitamin C treatment

Starting on day 105 of gestation, vitamin C (Ascorbate; A-5960; Sigma Chemicals, UK; 1.14 mmol/kg/day dissolved in 0.6 ml/kg saline and administered as a slow IV bolus injection) or saline vehicle (0.6 ml/kg slow IV bolus injection) were administered every day to the mothers at approximately 09:00. The dose of vitamin C used in this study was derived from previous studies in sheep pregnancy in our laboratory, which achieved elevations in circulating ascorbate concentrations within the required range for vitamin C to compete effectively in vivo with NO in ovine pregnancy [33,34,37].

### Blood sampling regimen and analysis

Samples of descending aortic maternal blood (0.3 ml) were taken daily for measurement of maternal blood gases and pH and hemoglobin concentration, as previously described [33,34,39,40]. Chamber oxygenation and maternal blood gases and pH were recorded at 104, 105, and 106 dGA then as summary averages of the preceding 5 days for 110, 115, 120, 125, 130, and 135 days. For all data, baseline for each animal was calculated as the average values of 104 and 105 days. On days 104, 105, 106, and 110 of gestation, and every 5 days after that, an additional 9 ml of maternal blood was taken and divided into tubes containing the anticoagulant EDTA or heparin. Samples were spun in a centrifuge for 5 minutes at 1000 x g and 4°C, after which the plasma was aliquoted into storage tubes and immediately frozen for subsequent analysis.

### Post mortem of ewes with fetuses

At 138 dGA, pregant ewes with male fetuses were transferred from the hypoxic chambers to the post mortem laboratory wearing a respiratory hood providing the same hypoxic mixture. Under hypoxic conditions, ewes and their fetuses were humanely killed by overdose of sodium pentobarbitone (0.4 ml.kg^−1^ IV Pentoject; Animal Ltd, York, UK) and the fetus exteriorized by Cesarean section. An 8-ml sample of fetal blood was taken from the umbilical artery using a syringe with an attached needle, divided into tubes containing the anticoagulant EDTA or heparin, then spun in a centrifuge for 5 minutes at 1000 x g and 4°C. The plasma was aliquoted into storage tubes and immediately frozen for subsequent analysis. Fetal crown rump length, biparietal diameter, and hind limb lengths were then determined. The upper hind limb comprised the length of the femur, the middle hind limb ranged between the patella and tuber calcis, and the lower hind limb ranged between the tuber calcis and the tips of the phalanges. Remaining fetal organs were dissected, weighed, and immediately frozen for subsequent analysis.

### Maternal and fetal blood and plasma analysis

The maternal arterial blood P_50_, the PO_2_ at which 50% of the maternal hemoglobin is saturated with oxygen, was calculated using the average of the maternal values for PO_2_ and Sat Hb from day 115–135 of gestation, according to the Hill equation [87]. Maternal and fetal plasma concentrations of NOx species (NO_2_^−^ and NO_3_^−^) were determined by a commercially available assay kit (Caymen Chemical, USA, Cat No. 780001) according to the manufacturer’s instructions. A standard curve was plotted in Excel using a straight line fit, allowing NOx concentrations to be calculated for each sample. The inter- and intra-assay coefficients of variation were 3.4% and 2.7%, respectively, and the lower limit of detection of the assay was 2.5 μmol/L. Maternal plasma concentrations of ascorbic acid were measured by a fluorimetric technique using a centrifugal analyzer with a fluorescence attachment, according to the method of Vuilleumier and Keck [88], in collaboration with the Core Biochemical Assay Laboratory, Cambridge, UK. The interassay coefficients of variation were 7.9% at 27.1 μmol/L and 5.0% at 89.7 μmol/L. The lower limit of detection of the assay was 10 μmol/L. Fetal plasma concentrations of total L-homocysteine were measured using a commercially available enzyme immunoassay kit (Axis-Shield diagnostics Ltd., UK, Cat No. FHCY100), in collaboration with the Core Biochemical Assay Laboratory, Cambridge, UK. The inter- and intra-assay coefficients of variation for a 6.1 μmol/L sample were 2% and 8%, respectively, and the lower limit of detection of the assay was 1.0 μmol/L. Fetal plasma cortisol concentrations were measured using a commercially available ELISA kit (IBL international, Germany, Cat No. RE52061), according to the manufacturer’s instructions. The lower limit of detection of the assay was 2.46 ng/mL. The cross-reactivity of the antiserum with other cortisol-related compounds was 4.2% cortisone, 1.4% corticosterone, 0.4% progesterone, and 7.0% deoxycortisol.

### Fetal liver oxidative stress analysis

The expression of 3-NT and SOD and the activity of catalase in frozen, powdered, fetal right liver lobe samples were determined by commercial assay kits (3-NT: ab116691, Abcam, Cambridge, UK, AMS biotechnology, Abington, UK, SOD: Sigma-Aldrich, Catalase: 707002), according to the manufacturer’s instructions.

### Placental analysis

Placentomes were classified into 4 categories by their gross morphological appearance, according to Vatnick and colleagues [89]. Following classification, the individual types were counted and weighed.

### Postnatal care of offspring

At 138 dGA, pregnant ewes carrying female fetuses were transferred from the hypoxic chambers to individual pens in a barn with a 12:12-hour light–dark cycle, where they were returned to normoxic conditions. Ewes were allowed to deliver naturally and remained with their offspring until weaning. Newborn lambs were weighed within 12 hours of birth and then daily for the first week of life. Thereafter, body weight was recorded at 2 weeks of age, 30 days of age, and then at subsequent 30-day intervals until they were 9 months old.

### Adult offspring surgery

At 265 ± 5 days (9 months) of age, when sheep are sexually mature and classified as young adults, the female offspring were surgically instrumented under general anesthesia with vascular catheters and a femoral artery flow probe. In brief, food but not water was withdrawn 10–15 hours before surgery in order to minimize the risk of bloat and regurgitation of the reticulo-rumen contents. On the day of surgery, anesthesia was induced by injection of Alfaxan (1.5–2.5 ml/kg IV alfaxalone; Jurox Ltd., Worcestershire, UK) into the jugular vein and maintained by spontaneous inhalation of 1.5% isoflurane in 60:40 O_2_:N_2_O (2 L/min; IsoFlo; Abbott laboratories Ltd., Berkshire, UK). Surgery was performed under aseptic conditions. An incision, approximately 3.5 cm long, was made in the medial surface of each of the hind limbs in order to expose the femoral artery and vein, which were catheterized as before. On the contra-lateral hind limb, a 4SB Transonic flow probe (Transonic Systems Inc., Ithaca, New York, USA) was placed around the main femoral artery for measurement of femoral blood flow. Catheters and the flow probe lead were then tunneled subcutaneously on their respective sides of the body and exteriorized via keyhole incisions made in the animal’s flanks. All skin incisions were then sutured closed and plastic pouches were sewn onto each flank to house the exteriorized catheters and flow probe. Following surgery, lambs were housed in individual floor pens with a 12:12-hour light–dark cycle with free access to hay and water. Antibiotics (30 ml/kg IM procaine benzylpenicillin; Depocillin; Intervet UK Ltd., Milton Keynes, UK) were administered daily to the lamb for 5 days following surgery, and catheters were flushed daily with heparinised saline (100 i.u/ml heparin, 0.9% NaCl). Following at least 5 days of postoperative recovery, basal and stimulated cardiovascular function was assessed in vivo. Post mortem, third-order femoral arteries (internal diameter < 300μm) were isolated and reactivity was determined via in vitro wire myography.

### Adult offspring basal and stimulated in vivo cardiovascular function

Experimental protocols were performed following at least 5 days of postoperative recovery. On the morning of an experiment, ewe lambs were moved into a metabolic crate where their arterial catheter was connected to a sterile pressure transducer (Argon Division, Maxxim Medical, Athens, Texas, USA) and their flow probe to a Transonic flow meter (T206; Transonics Systems Inc., Ithaca, NY, USA). On each experimental day, lambs were allowed to acclimatize for 2–3 hours before commencing recordings. Basal cardiovascular variables were recorded continuously for 4–6 hours. A custom-built Data Acquisition System (Maastricht-Programmable AcQuisition system, M-PAQ and IDEEQ software, Maastricht Instruments, the Netherlands; 1000-Hz sample rate) was used to record arterial blood pressure. Blood flow signals from the Transonic flow meter were also directly recorded by the IDEEQ software, and heart rate was calculated continuously online by the program using the femoral blood flow or systolic pulse as a trigger.

On a separate day, stimulated cardiovascular function in the ewe lambs was determined by measuring the change in mean arterial pressure, heart rate, and femoral blood flow in response to increasing bolus doses of the vasoconstrictors PE (0.25, 0.5, 2, 4, 8, 16, 32, and 64 μg/kg IA, diluted in 1 ml dH2O; L-phenylephrine; P-6126; Sigma Chemicals, UK); and Ang II (5, 10, 20, 40, 80, 160, and 320 ng/kg IA, diluted in 1ml dH_2_O; MP Biomedicals, California, USA) or in response to increasing doses of the NO donor SNP (0.625, 1.25, 2.5, 5, and 10 μg/kg IA, diluted in 1 ml dH2O; S-0501; Sigma Chemicals, UK). Suitable dose ranges were derived from pilot experiments and previous studies in the literature [90,91]. All doses were administered in a random order. On a separate day, the change in femoral vascular resistance was also determined after intravenous treatment with the NO synthase inhibitor L-NAME (100 mg/kg; Cayman Chemicals, Cambridge, UK).

### Post mortem of adult offspring

At the end of all experimental procedures, ewe lambs underwent euthanasia with a lethal overdose of sodium pentobarbital administered to the indwelling venous catheter (200 ml/kg IV Pentoject; Animalcare Ltd., York, UK). The brain, heart, and liver were dissected and weighed. The brain was further dissected into the midbrain plus diencephalon, cerebellum, and right and left hemispheres for compartmental weighing and storage of each section.

### In vitro wire myography of adult offspring femoral arteries

Third-order femoral arteries (internal diameter < 300μm) were isolated and placed in physiologic buffer solution (PBS). A segment of approximately 2 mm in length was cut and threaded with two 40-μm diameter stainless steel wires. The vessel segment was then mounted on a wire myograph (Multi Wire Myograph System 610M; DMT, Denmark), while bathed in Krebs solution (mM: NaCl 118.5, KCl 4.75, MgSO_4_ 7, H_2_O 1.2, KH_2_PO_4_ 1.2, NaHCO_3_ 25.0, CaCl_2_ 2.5, and glucose 5.5; Sigma) and constantly exposed to a gas mixture of 5% CO_2_ and 95% O_2_ at 37°C in the myograph chamber. Following a 30-minute equilibration period, the vessel was stretched in a stepwise manner to a standardized tension equivalent to physiologic transmural pressure. Following a 20-minute equilibration period, all vessels were then precontracted with PE (10^−5^ M) before assessing endothelium-dependent vasodilator responses to different concentrations of methacholine (MetCh; 10^−9^ to 10^−4^ M; Sigma Aldrich). To determine the relative contribution of endogenous NO, EDHF, and prostanoid to endothelium-dependent relaxation, concentration-response curves to MetCh were also generated following incubation for 10 minutes with L-NAME (10^−5^ M; Sigma Aldrich) and L-NAME plus indomethacin (10^−6^ M; Sigma Aldrich), as previously reported [92]. Vessels were washed repeatedly with Krebs solution and allowed to equilibrate for at least 20 minutes between different concentration-response curves.

### Data analysis and interpretation

For the in vivo cardiovascular experiments at adulthood, variables representing basal cardiovascular function represent the average of the 4–6-hour recording period, which was always during the same time of the day. Femoral vascular resistance (FVR) and femoral vascular conductance (FVC) were calculated by applying Ohm’s Law to the circulation, using the following equations, for which ABP is the arterial blood pressure and FBF is femoral blood flow: FVR = ABP/FBF and FVC = FBF/ABP. An increase in vascular resistance signifies a reduction in blood flow greater than can be accounted by a reduction in arterial blood pressure, therefore active vasoconstriction. An increase in vascular conductance signifies an increase in blood flow greater than can be accounted by an increase in arterial blood pressure, therefore active vasodilatation.

For the in vivo dose-response experiments at adulthood, maximal changes from baseline in cardiovascular variables were recorded for each dose. The baseline was taken as the preceding 1–2 minutes of stable recording before each dose was given. After each dose, cardiovascular variables were allowed to return to baseline and remain stable for at least 2 minutes before preparing for the next dose.

For the in vitro wire myography experiments at adulthood, femoral arterial responses were analysed using Prism (v.5.0, GraphPad software). Concentration-response curves were analysed using a sigmoidal fit curve. The maximal vessel relaxation (percent Rmax) was expressed as percentage of the contraction induced by PE. The contribution of NO-dependent mechanisms to the relaxation induced by MetCh was calculated by subtracting the area above the curve (AAC) for MetCh–the AAC for MetCh + L-NAME. The contribution of NO-independent mechanisms was the AAC for MetCh + L-NAME. The contribution of prostanoid to the relaxation induced by MetCh was calculated as the AAC for MetCh + L-NAME–the AAC for MetCh + L-NAME + indomethacin. The remaining AAC following MetCh + L-NAME + indomethacin was taken as EDHF [92].

For all in vivo and ex vivo experiments, data are expressed as the mean ± SEM. All variables were assessed as appropriate either using two-way ANOVA comparing the interactions between oxygenation and treatment or two-way ANOVA with repeated measures comparing the effects of group and dose or time. Where a significant effect was indicated, Tukey’s posthoc test was used to isolate the statistical differences (Sigma-Stat 3.5; Chicago, IL, USA and GraphPad Prism 6). For all comparisons, statistical significance was accepted when *P* < 0.05.

## Supporting information

S1 Data Raw data. Complying with data policy, Excel spreadsheet files are provided with the underlying numerical data points for all graphs contained within the manuscript.
(XLSX)

S1 Fig Placental measurements. Measurements at 138 dGA: A, total placentome weight; B, placentome distribution; and C, fetal:placentome weight ratio. Values are mean ± SEM. Groups are N (open symbols, *n* = 12), H (grey symbols, *n* = 8), HC (red symbols, *n* = 9), and NC (blue symbols, *n* = 9). There are no significant differences between groups. dGA, days of gestation; H, hypoxia; HC, hypoxia with vitamin C, N, normoxia; NC, normoxia with vitamin C.
(TIF)

## Abbreviations

AngII: angiotensin II
ANOVA: analysis of variance
AAC: area above the curve
AWERB: Animal Welfare and Ethical Review Board
BMI: body mass index
BPD: biparietal diameter
CRL: crown rump length
dGA: days of gestation
EDHF: endothelium-derived hyperpolarizing factor
[Hb]: hemoglobin concentration
HCO_3_^−^: bicarbonate
Hcy: homocysteine
INTAPP: international trial of antioxidants in the prevention of preeclampsia
LLL: lower limb length
L-NAME: N(ω)-nitro-L-arginine methyl ester
MetCh: methacholine
NO: nitric oxide
NOx: plasma concentrations of total NO_3_^−^ + NO_2_^−^
O_2_: oxygen
P_50_: PaO_2_ value at which 50% of hemoglobin is saturated with oxygen
PaCO_2_: arterial blood partial pressure of carbon dioxide
PaO_2_: arterial blood partial pressure of oxygen
PBS: physiologic buffer solution
PE: phenylephrine
PG: prostaglandin
PM: post mortem
Sat Hb: arterial blood percentage saturation of hemoglobin with oxygen
ROS: reactive oxygen species
SEM: standard error of the mean
SNP: sodium nitroprusside
SOD: superoxide dismutase
percent Rmax: maximal vessel relaxation
VIP: vitamins in preeclampsia
