# Supplementary figures and images for: Intervention against hypertension in the next generation programmed by developmental hypoxia
Source: PLoS Biol. 2019 Jan 22;17(1):e2006552. doi: 10.1371/journal.pbio.2006552 (PMC6342530; doi:10.1371/journal.pbio.2006552)

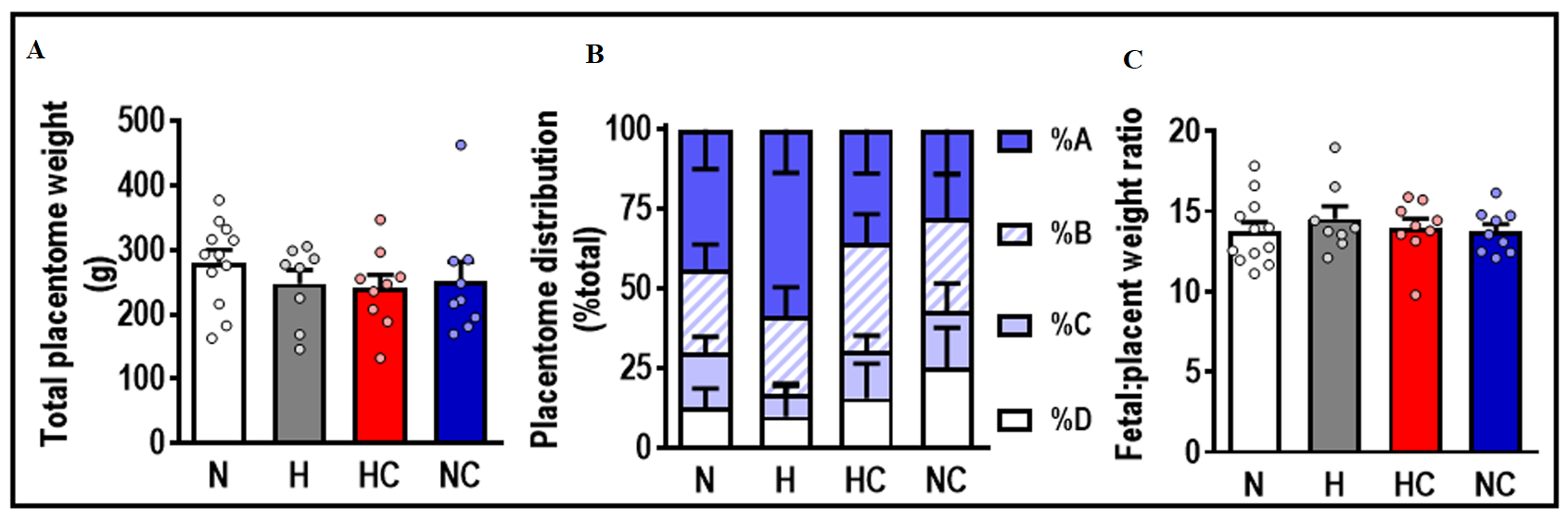

Supplement: S1 Fig — Measurements at 138 dGA: A, total placentome weight; B, placentome distribution; and C, fetal:placentome weight ratio. Values are mean ± SEM. Groups are N (open symbols, n = 12), H (grey symbols, n = 8), HC (red symbols, n = 9), and NC (blue symbols, n = 9). There are no significant differences between groups. dGA, days of gestation; H, hypoxia; HC, hypoxia with vitamin C, N, normoxia; NC, normoxia with vitamin C. (TIF) [file pbio.2006552.s002.tif]
